# The Coaching on Lifestyle (CooL) Intervention for Overweight and Obesity: A Longitudinal Study into Participants’ Lifestyle Changes

**DOI:** 10.3390/ijerph15040680

**Published:** 2018-04-04

**Authors:** Celeste van Rinsum, Sanne Gerards, Geert Rutten, Nicole Philippens, Ester Janssen, Bjorn Winkens, Ien van de Goor, Stef Kremers

**Affiliations:** 1 Department of Health Promotion, NUTRIM School of Nutrition and Translational Research in Metabolism, Maastricht University, P.O. Box 616, 6200 MD Maastricht, The Netherlands; sanne.gerards@maastrichtuniversity.nl (S.G.); n.philippens@maastrichtuniversity.nl (N.P.); e.janssen@maastrichtuniversity.nl (E.J.); s.kremers@maastrichtuniversity.nl (S.K.); 2 Faculty of Humanities and Sciences, University College Venlo, Maastricht University, P.O. Box 8, 5900 AA Venlo, The Netherlands; geert.rutten@maastrichtuniversity.nl; 3 Department of Methodology and Statistics, CAPHRI Care and Public Health Research Institute, Maastricht University, P.O. Box 616, 6200 MD Maastricht, The Netherlands; bjorn.winkens@maastrichtuniversity.nl; 4 Department Tranzo, Tilburg School of Social and Behavioral Sciences, Tilburg University, P.O. Box 90153, 5000 LE Tilburg, The Netherlands; l.vandegoor@uvt.nl

**Keywords:** lifestyle, coaching, overweight, obesity, physical activity, nutrition/diet, behaviour change, combined lifestyle intervention

## Abstract

Combined lifestyle interventions (CLIs) can be effective in reducing weight and improving lifestyle-related behaviours but it is unclear how CLIs can best be implemented in practice in order to achieve sustained lifestyle changes. The Coaching on Lifestyle programme (CooL) is a CLI in the Netherlands, in which professional lifestyle coaches counsel adults and children (and/or their parents) who are obese or at high risk of obesity to achieve a sustained healthier lifestyle. The CooL intervention consists of group and individual sessions addressing the topics of physical activity, dietary behaviours, sleep and stress. Our longitudinal one-group pre-post study aimed to identify lifestyle changes among participants (adults, children and their parents) at 8 and 18 months after initiation. We assessed constructs ranging from motivation and behaviour-specific cognitions to behaviours and health outcomes. Positive and sustained changes among adults were found regarding perceived autonomy, motivation, perceived barriers, lifestyle behaviours, quality of life and weight. Among children and their parents, few improvements were found regarding behaviours and quality of life. CooL has been successful in coaching adult participants towards sustained behavioural change during the intervention period. Mixed results and smaller effect sizes were found for children and their parents.

## 1. Introduction

Worldwide, the prevalence of overweight and obesity is still increasing [1,2]. In The Netherlands, 50.2% of the adult population are overweight, 14.5% of whom are obese [3]. The number of children with overweight and obesity in particular has rapidly increased in recent decades [4]. Currently, 13.4% of the children in The Netherlands are overweight and 2.7% are obese. Overweight is caused by an imbalance between energy intake and energy expenditure [5]. Primary factors that may lead to a chronically disturbed energy balance include insufficient levels of physical activity, too much sedentary behaviour [6] and too much high-energy dietary intake [7]. This imbalance can also be influenced by sleep, since sleep deprivation may lead to a decrease in physical activity and an increase in energy intake due to disturbed hormone levels [8,9]. 

Combined lifestyle interventions (CLIs) support people who are overweight or obese in initiating and maintaining healthier lifestyle behaviours, by increasing physical activity and improving dietary behaviours. CLIs include physical activity, dietary and behavioural components [10] and are typically carried out by general practitioners, practice nurses, physiotherapists, psychologists and/or dieticians [11]. These interventions have been shown to be effective in terms of weight reduction and health improvements compared with standard care or drug treatment [10,12]. Lifestyle effects of CLIs and other types of weight loss interventions, however, have been shown to be rather short-term; relapses are common in long-term weight loss [13]. 

The main causes of relapse are that it is not easy to incorporate sustainable lifestyle changes into daily behaviour patterns and that intervention success depends heavily on the quality and quantity of external stimuli from professionals. Motivation, as well as well-being and resilience, are strong predictors of sustained healthy lifestyle behaviours and weight loss [13]. In addition, previous CLI studies identified undesirable implementation issues: lack of multidisciplinary collaboration, insufficient coaching skills among primary care professionals, insufficient time to optimize coaching and high intervention costs [14,15]. 

In comparison to coaching adult participants, supporting lifestyle changes among overweight children may even be more complex. Since parents play a key role in determining their children’s lifestyle behaviour [16], both parents as well as their children are advised to participate in CLIs. However, parents often do not see their child’s weight as a cause for concern [17], which decreases the likelihood that these families will participate in CLIs. In addition, the interplay between multiple contextual family-based factors (parenting, broken families, lack of resources) may undermine potential intervention effects [16]. 

Insights into implementation issues obtained from previous intervention studies on both adults and children in The Netherlands [14,18,19,20] have led to the design of the Coaching on Lifestyle (CooL) intervention for people who are overweight or obese [21]. The present paper discusses the results of the CooL intervention in a longitudinal one-group pre-post study using validated questionnaires and objective data. The study investigated the motivational, behavioural, quality of life and weight changes achieved by the CooL participants (i.e., adults and children) during the intervention period and in the longer term. 

## 2. Methods

A detailed description of the study protocol can be found elsewhere [21]. Below, we provide more details about the CooL intervention, followed by the study design and methods. This study was registered in a trial register (Dutch Trial Register: NTR6208) and it was exempt from review by a research ethics committee (METC 14-5-021), as it does not fall within the scope of the Dutch Medical Research Involving Human Subjects Act (Central Committee on Research Involving Human Subjects (CCMO), 2015).

### 2.1. CooL: The Coaching on Lifestyle Intervention

CooL is a CLI in The Netherlands, in which professional lifestyle coaches counsel adults and children (and/or their parents) who are obese or at high risk of obesity to help them achieve a sustained healthier lifestyle. The lifestyle coaches who took part in the present study had completed a post-graduate lifestyle coaching education programme. Health coaching or lifestyle coaching, although relatively new in both practice and research [22], is a promising approach to achieve positive behavioural and health outcome changes [23,24,25].

Lifestyle coaching entails a client-oriented approach. The starting-point is the current lifestyle and the associated healthy and unhealthy habits of the participant. During the process of changing their lifestyle, the participants remain in charge of their intended (behavioural) goals. Unhealthy behaviours (mainly concerning physical activity, nutrition, sleep, stress management and relaxation) are identified and throughout the coaching sessions each participant is stimulated to replace unhealthy routines by healthier alternatives. The lifestyle coaches support participants in their change process by helping them to identify their unhealthy as well as their healthy habits and underlying processes. Furthermore, they stimulate the participants to take responsibility for their own behaviour and to identify challenging but achievable action steps for each of the above behaviours in order to set feasible and sustainable goals that will improve their long-term health. The lifestyle coaches’ role includes listening, supporting, motivating and providing feedback to the participants as well as providing background information on health topics and behavioural change [24].

The CooL programme consists of 8 group sessions and 4 to a maximum of 10 individual sessions targeting physical activity, dietary behaviours, sleep and stress. The programme as a whole lasts approximately 8 to 10 months. Three different basic programmes focus on three age groups: primary school children (aged 4 to 12 years), adolescents (aged 12 to 18 years) and adults (aged 18 years and older). In the children’s and adolescents’ programmes, parents participate together with their child.

Various evidence-based techniques and approaches are incorporated in the basic programmes (see Appendix A, Table A1), which are similar to other interventions [26,27]. In addition to the basic programmes, coaches can, if required, assess the needs of each participant and the appropriateness of being included in the relapse prevention intervention (group and individual sessions) or the additional intervention (individual sessions). These supplementary programmes were set up to prevent relapse and to maintain the healthy behaviours in the longer term.

### 2.2. Design and Study Population

In this one-group pre-post study, the lifestyle changes of the CooL participants were monitored over time. The target population of this intervention consisted of Dutch-speaking individuals living in The Netherlands, aged 4 years and older, who were obese (BMI ≥ 30) or at high risk of obesity (i.e., were overweight (BMI ≥ 25) and at increased risk of cardiovascular diseases or type 2 diabetes mellitus) [28,29,30]. The exclusion criterion was being unable to function in a group (e.g., because of behavioural disorders).

Adults were often referred by their general practitioner or practice nurse in three different regions (Oosterhout and its region, Parkstad and Tilburg) within two provinces of the southern part of The Netherlands. The recruitment of children mostly took place via referral by Youth Health Care (YHC) professionals, schools (such as those taking part in the “Healthy Elementary School of the Future” programme [31]), pedagogical workers and paediatricians in two regions (‘s-Hertogenbosch and Breda) in one of the same two provinces. Participants could also sign up for the intervention themselves. These participants were instructed to consult their health care provider (e.g., general practitioner) to check whether they met the inclusion criteria. All participants were asked to sign an informed consent form when they started in the study. Both parents were asked to sign for informed consent and in case of adolescents (12–18 years), also the child had to sign an informed consent form.

### 2.3. Measurements

#### 2.3.1. Questionnaires

The participants completed three questionnaires: at baseline (T0); immediately after the intervention, which was approximately 8 to 10 months after baseline (T1); and 18 months after baseline (T2). The questionnaires were adapted to the three different groups: the older children (aged 10 years and older) and their parents both filled in their own questionnaires, while the adult participants (18 years and older) received a different version of the questionnaire. Children younger than 10 years were considered too young to fill out a questionnaire. The constructs measured in the questionnaires were based on the theoretical framework of this study [21], which consists of psychological needs, parenting, motivation, behaviour-specific cognitions (e.g., barriers) and energy balance-related behaviours, which were assumed to produce the health outcomes (i.e., quality of life and BMI). Where possible, validated questionnaires were used (see Appendix A, Table A2 and Table A3 for an overview and reliability indices). The attendance lists, filled out by the lifestyle coaches, provided insights in the attendance rates and drop-out.

#### 2.3.2. Body Weight

Body mass index (BMI) of adults was objectively measured by the practice nurses, general practitioner and/or researchers. When objective weight data were missing (14%), they were replaced by self-reported weight. The weight status was classified into five categories according to international guidelines [32]: normal weight (18.5 ≤ BMI ≤ 24.99 kg/m^2^), overweight (BMI ≥ 25 kg/m^2^), obesity (BMI ≥ 30 kg/m^2^), severe obesity (BMI ≥ 35 kg/m^2^) and morbid obesity (BMI ≥ 40 kg/m^2^). 

For children, we used objectively measured BMI, as assessed by YHC professionals, lifestyle coaches and/or researchers. When objective data were missing, we imputed this with the child’s weight as reported by the parent. The data were converted to BMI standardized for age and gender (i.e., BMI z-score) using the Dutch reference population [28,33]. Weight status was categorized using international cut-off values [34].

#### 2.3.3. Statistical Analyses

Numerical variables are presented as means (SD), while the number of participants (%) is used for categorical ones. Linear mixed model analysis was used to assess the changes over time, using an unstructured or compound symmetric (where appropriate) covariance structure for repeated measures. In addition, a random intercept at coach level was included to take the nesting of participants within coaches into account. The longitudinal trend was assessed by including time (categorical; T0, T1 and T2) in the fixed part of the model and was corrected for demographic characteristics such as age, gender, living situation, occupational status, highest completed education and BMI or BMIz (if BMI was not the outcome), if these characteristics were related to missing outcome values. All data from participants who did not drop out before the first group session were included in these analyses. Longitudinal effects were presented as corrected estimated mean difference in change scores from baseline, together with their corresponding 95% confidence intervals and *p*-values. 

Standardized effect sizes were also included, which were defined as (mean at T1 or T2—mean at T0)/standard deviation at T0. The effect sizes were categorized in accordance with Lipsey’s guidelines [35]: small effect (0–0.32), medium effect (0.33–0.55) and large effect (higher than 0.56). As a result of the large amount of outcome measures, we aimed to summarize the observed changes in a graph for illustrative purposes. For the adult population, the average effect sizes regarding psychological needs, motivation and barriers (‘motivation’), physical activity, dietary behaviours (‘behaviour’), weight and quality of life were plotted in a graph. For the children and adolescents, the average effect sizes of the general parenting and parenting practices were plotted together (‘parenting’), the motivation and behaviour-specific cognitions (‘motivation’) and the physical activity, dietary and sleeping behaviours (‘behaviour’).

All analyses were performed using IBM SPSS Statistics for Windows, version 23.0 [36]. A two-sided *p*-value ≤ 0.05 was considered statistically significant.

## 3. Results

### 3.1. Characteristics of Participating Adults

The pre-test questionnaire was filled in by 138 participating adults between 20th of May 2014 and 15th of February 2016. The adult participants consisted of an evenly divided group of men and women, whose mean age was 55.1 years (SD 10.1; see Table 1). In total, 86% of the participants were obese. Almost all (95%) participants had been born in The Netherlands, more than three quarters of participants had a low or intermediate educational level, more than half the participants did not have a steady job and most of them (71%) were living with a partner. 

### 3.2. Characteristics of Participating Children

Demographic characteristics of 31 children and 10 adolescents who participated (referred to below as “children”) are presented in Table 1. Children were nearly evenly divided in terms of gender. The average BMI z-score of children was 2.4 (SD 0.4). A large majority of the children who participated were obese (93%). Most parents were overweight too. The mean BMI of the mothers and fathers was 30.4 (SD 6.5) and 27.3 (SD 4.6), respectively. Almost all children were born in The Netherlands (97%) but 23% had a Turkish or Moroccan migration background. The majority of the mothers had an intermediate or lower educational level (73%) and the majority of the fathers had a low educational level (57%). In more than half of the families both parents had a job and most parents were living together. On average the families consisted of four persons.

### 3.3. Drop-Out and Attendance

Among the adult participants, 13% (*n* = 18) dropped out of the intervention. There was a loss to follow-up regarding measured weight of 20% at T1 and 23% at T2 (included *n* = 110 and *n* = 106, respectively). Regarding the questionnaires, the loss to follow-up was 33% at T1 (*n* = 93) and 45% at T2 (*n* = 76). Of the demographic characteristics, only gender was significantly different in the adult drop-out group compared with the rest, as more women than men dropped out (*p* = 0.023). Among the children, 7% (*n* = 3) dropped out of the intervention. The loss to follow-up regarding measured weight was 22% at T1 and 29% at T2 (*n* = 32 and *n* = 29, respectively), while loss to follow-up regarding the questionnaires was 41% at T1 and 49% at T2 (*n* = 24 and *n* = 21, respectively). For the children, there were no significant demographic predictors of loss to follow-up.

The total amount of sessions in the protocol for adults consisted of 8 groups sessions and 3.5 individual hours. The adults participated on average in 5.9 (±1.9) group sessions and had 3.2 (±0.7) h of individual counselling from their lifestyle coach. Their average total intervention period was 219.9 (±76.0) days. The protocol for children and/or their parents consisted of 8 group sessions and 7 h of individual counselling. The children and/or their parents attended on average in 4.6 (±2.6), group sessions and they had 5.4 (±1.4) h of individual sessions. They participated in the intervention for a period 283.0 (±103.0) days. 

### 3.4. Changes among the Adult Participants

**Figure 1:**
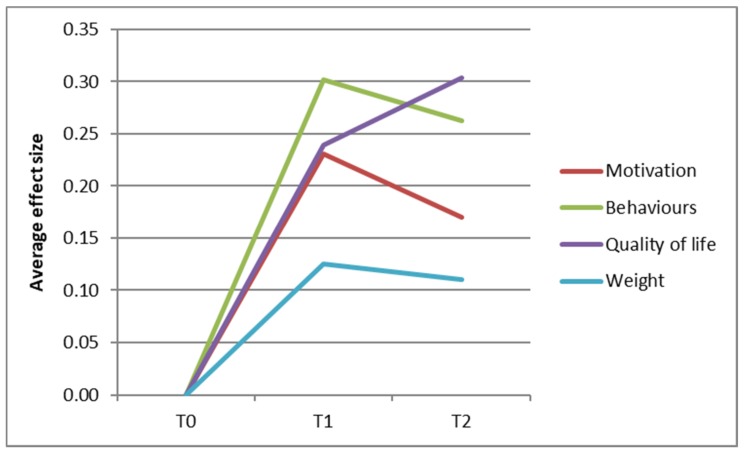
Overall changes among the adults regarding motivation, behaviours, quality of life and weight.

Figure 1 offers a visual summary of all the changes among the adult participants regarding motivation, behaviours, quality of life and weight. A positive change in effect size corresponds to a change in a healthier direction. All four components were increased at T1, followed by a slight decrease (quality of motivation, behaviour and weight) or further increase (quality of life) at T2. All aggregated effect sizes can be categorized as small (0–0.32).

#### 3.4.1. Psychological Needs

Perceived autonomy increased significantly, with a small effect size, after the intervention, by 0.2 points on a 5-point Likert scale, which was maintained in the longer term at T2 with a medium effect size (Table 2). This means that the participants reported a higher sense of volition and willingness to engage in physical activity, compared to baseline.

#### 3.4.2. Motivation for Physical Activity and Healthy Diet

External motivational regulation had decreased significantly and both integrated regulation and intrinsic motivation had increased significantly at post-test. This applies to the motivation for physical activity and that for a healthy diet, with some exceptions at T2. Introjected regulation for healthy diet had decreased significantly at the two follow-up moments. Overall, participants were more autonomously motivated (e.g., exercising because it is fun or eating a healthy diet because it is part of who you are) after the intervention, with small to medium effect sizes and had less controlled motivation (exercising because others say you should) for physical activity at T2 and for healthy diet at the post-test.

#### 3.4.3. Behaviour-Specific Barriers

Participants perceived significantly fewer barriers, such as lack of time, lack of good equipment or lack of discipline, to being physically active and to eating a healthy diet at T1 and T2 compared with the pre-test, with medium effect sizes. 

#### 3.4.4. Energy Balance-Related Behaviours

Participants reported sitting significantly less after the intervention compared with the pre-test (−123.2 min a day), with a large effect size. They also engaged in more moderate-intensity and vigorous-intensity activity (medium effect size), with the largest increase at T2. Changes in dietary behaviour were in the desired direction. Participants reported eating breakfast more often and eating more fruits and consuming less fruit juice, sugar-sweetened beverages and unhealthy snacks (small to medium effect sizes). Changes regarding sugar-sweetened beverages and consumption of unhealthy snacks were maintained in the longer term.

#### 3.4.5. Quality of Life and BMI

The participants reported a significantly better quality of life for the dimensions of self-care, pain and anxiety (medium effect size) at post-test. The pain and anxiety dimensions remained improved at T2. This led to better total quality of life scores at T1 and T2. Health in general and perceived health on the day they completed the questionnaire, which are also quality of life indicators, had increased at T2 and health in general had also increased at T1 (medium effect sizes).

The participants lost an average of 2.3 kg immediately after completing the CooL intervention, which corresponds to 0.8 BMI points. At T2, the average weight loss was still 1.8 kg, compared to the pre-test. In total, 63.2% of the participants had lost weight at T1; 25.0% of the participants had lost more than 5% body weight; and 44.3% of the participants had lost more than 2% body weight (data not shown). At T2, 61.8% of the participants had lost weight compared to baseline, while 21.6% had lost more than 5% weight and 39.6% had lost more than 2% of their original weight.

### 3.5. Changes among the Children Participants

**Figure 2:**
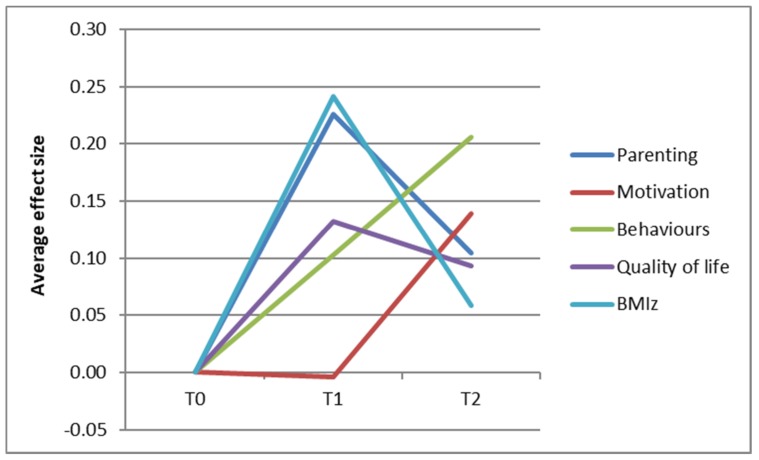
Overall changes among the parents and children regarding parenting, motivation, behaviours, quality of life and BMIz.

Figure 2 shows all the healthy changes among the parents and children regarding parenting, motivation, behaviours, quality of life and BMIz. All aggregated effect sizes clustered around 0.10 at T2 (small effect sizes), except for the behaviours (with an average effect size of 0.20).

#### 3.5.1. General Parenting and Parenting Practices

At T1, parents experienced fewer problems with their child’s behaviour compared to the pre-test, with a small effect size (−0.5 on a 7-point Likert scale; Table 3). In addition, their self-efficacy as regards managing these problems had increased at T1, with a large effect size. After the programme, parents showed a healthier role model behaviour regarding healthy eating and being physically active in the presence of their child (medium to large effect sizes). This role modelling for healthy eating was maintained at T2. The children’s food environment had also improved at T1 and T2, with parents providing more healthy food for their children (medium to large effect sizes). In addition, their so-called “covert control” practices had also changed at T1 and T2, with medium effect sizes: parents more often tried to influence their children’s intake of unhealthy products in a way that was not directly visible to the child, for example by reducing the availability or visibility of unhealthy food products at home. 

#### 3.5.2. Motivation and Behaviour-Specific Cognitions

The children appeared not to be affected by the intervention as regards the motivational sub-scales for physical activity, since no statistically significant changes from baseline were detected. However, medium effect sizes were found regarding their intrinsic motivation at T1 and their introject regulation at T2. Children’s self-reported attitude towards fruit showed a significant negative change after the intervention compared with the pre-test (medium effect size). No significant changes were observed regarding enjoyment of physical activity, habit strength for physical activity and healthy eating, or regarding self-efficacy. Medium effect sizes were found at T1 or T2 for habit strength for playing outside and eating and self-efficacy regarding playing outside.

#### 3.5.3. Energy Balance-Related Behaviours

Immediately after completing the intervention, as well as in the longer term, the children were spending significantly less time watching television compared with the pre-test (−2.1 and −4.5 h per week, respectively) as reported by their parents, with small to large effect sizes. A large effect size was found regarding the sports behaviour reported by the children at T2. The parents reported that their child was eating more vegetables and was drinking water more often and was drinking sugar-sweetened beverages and fruit juices less often, at T1 or T2, with small to large effect sizes. Medium to large effect sizes were found at T1 for eating fruit and unhealthy snacks and drinking diet beverages. Regarding the other physical activity and dietary behaviours, small and non-significant changes were found. At the pre-test, 23% of the children suffered from sleeping problems (data not shown). The number of sleeping problems had not significantly decreased after the intervention.

#### 3.5.4. Quality of Life and BMIz

At post-test, the physical comfort dimension and the overall quality of life score had improved significantly compared to the pre-test (small effect sizes). Parents reported that their child was able to move better and with greater ease. We found medium effect sizes for the family relations dimension at T1 and the physical comfort dimension at T2. On average, children did not differ significantly in terms of their BMIz after the intervention. 

## 4. Discussion

In this study we monitored the motivational, behavioural and weight changes among adults and children with obesity or overweight participating in the CooL intervention over a period of 1.5 years. Among the adult participants, we found significant improvements regarding perceived autonomy, motivational regulation for physical activity and healthy diet, perceived barriers to engaging in physical activity and eating a healthy diet, physical activity, healthy dietary behaviours and quality of life. Adults lost an average of 2.3 kg of body weight, which was largely maintained approximately one year later. These findings are relatively favourable compared to what was found in previous studies on implemented “real-life” CLIs [18,37,38,39,40], in which the body weight change varied between losses of 0.6 kg [41] and 3.0 kg over a study period of one year or longer [42,43]. 

The children in our study, however, showed less significant changes and smaller effect sizes. We found significant improvements regarding the time spent watching television, dietary behaviours, physical comfort and overall quality of life score. In contrast, children developed a significantly more negative attitude towards fruit consumption. They did not significantly change their BMIz score. In general, there were no significant changes regarding the children’s physical activity, motivation, habit formation or weight. However, medium to large effects sizes were found for some motivational, behavioural and quality of life outcomes. Their parents, however, perceived significantly less problems with their child’s behaviour, they increased their self-efficacy as regards addressing these issues and they improved their role modelling regarding healthy eating and physical activity, food environment and covert control. 

One of the main aspects of the CooL approach is an autonomy-supportive coaching style, which has been shown to predict a shift towards more autonomous types of motivation among participants [44]. Motivational regulation for physical activity and healthy diet is a predictor of changing and maintaining healthier behaviour [7,45]. Among our adult population we did indeed observe some changes towards more autonomous types of motivation. The adults showed more autonomous types of motivation at T1. Although this was not fully maintained at T2, the moderate and vigorous physical activity scores had even increased at T2. This result may imply that improved quality of motivation functions as a mediator to sustained behavioural change. An improved quality of motivation may be needed to induce behavioural changes, after which other habitual or routine processes play a role in maintaining these changes [46,47,48].

CLIs are typically carried out by multiple professionals, with a strong basis in primary care (e.g., general practices). Ours was the first study that investigated the effects of a lifestyle coaching intervention within The Netherlands in which a lifestyle coach was positioned as the central health professional, outside the traditional primary care chain. One of the outcomes from earlier studies [15] was that the participating professionals were undereducated and lacked resources (including sufficient time) to optimally counsel participants regarding their lifestyle. The post-graduated lifestyle coaches in our study showed that they were able to sustainably change the lifestyle of their participants using a step-by-step approach focusing on an autonomy-supportive coaching style that combines client-tailored individual sessions with more general group sessions. Note that lifestyle coaches in real-life settings need to be highly adaptive to the health care context, client profiles and public health systems. 

A clear difference between the adults and children was observed regarding maintained changes. Firstly, in comparison to adults, it proved to be harder to include children (and their parents) in the intervention. Recruitment issues have been encountered previously in childhood obesity interventions [49]. Reasons for this include denial of the problem by parents and resistance of parents towards discussing weight issues, as well as professionals who feel unable (through lack of skills or self-efficacy) to motivate parents to participate in obesity interventions and inhibiting societal norms regarding weight and participation in child overweight programmes. The adult participant recruitment was also insufficient, since the study sample was smaller than expected. Apart from recruitment issues, it may also be more difficult to achieve behavioural change in children, since they often have complex family situations and rooted routines. Another reason can be the complexity of the child’s obesity problem. When a child or its family are facing additional problems, it might not be the best time to start a lifestyle intervention. In such situations other, more urgent, problems (e.g., safety and poverty) need to be tackled first, after which other needs (e.g., losing weight) can be discussed [8]. This is a topic for which the coaches should be better trained, or for which coaches could involve with other health professionals more often.

### Strengths and Limitations

Strengths of this study include its real-life setting, longitudinal design and the use of validated questionnaires. The combination of multiple outcome variables provides a general overview of cognitive and behavioural changes among the participants. However, in order to shorten the questionnaires, not all the variables were included in every measurement. Another strength was that the group that has been exposed to this CooL intervention was geographically heterogeneous, as it has been implemented in multiple regions within The Netherlands. In addition, the study sample was representative of the Dutch population in terms of educational level and living situation. Note that a relatively large part of the study sample had a low or intermediate educational level. Most comparable studies have had difficulties recruiting this specific target population [40]. In our intervention the participants were recruited via the primary care system (i.e., general practices and YHC professionals), which has been reported to be a good strategy for including participants with a lower or intermediate educational level [40]. 

Some limitations have to be mentioned too. Including a control group in the design would have been beneficial in terms of making strong statements regarding the effectiveness of the intervention. However, the primary focus of this study was on the implementation process (i.e., following the implementation and changes among participants) [21], which reduced the need for a usual care control group. Previous studies on CLIs that did include a control group have shown relative stable patterns of behaviour and weight within the control group [50,51]. Furthermore, there is also a risk of social desirability in the answers regarding motivation, quality of life and behaviour.

Due to logistical issues, the post-test objective weight data were not measured at the exact same time as the questionnaires were completed. Therefore, we used a wider time frame (several months before and after the T1 or T2) to link these data to the T1 or T2 data. It is known that overweight or obese individuals underreport their dietary intake [8], height and weight [52,53]. In this study, the objectively measured weight and height were combined with the self-reported data to decrease the amount of missing values. This may have led to partially biased data. However, the degree of consistency between objective and self-reported weight data for adults was very high for the cases where we had access to both data sources (r = 0.985), which offers some confidence in the internal validity of these data. 

## 5. Conclusions

It can be concluded that the lifestyle coaches in this CooL intervention have been successful in coaching the adult participants who are obese or at high risk of obesity towards sustained behavioural change during the study period. Statistically significant changes and medium to large effect sizes among adults were found concerning cognitive and behavioural lifestyle factors and weight. For children and their parents, mixed results and smaller effect sizes were found. 

## Figures and Tables

**Table 1:** Demographics and weight-related characteristics of the participants.

Variable	Adult Participants (*n* = 138)		Child (*n* = 31) and Adolescent (*n* = 10) Participants	
	Mean (SD)	*N* (%)	Mean (SD)	*N* (%)
Gender				
Male		66 (47.8)		18 (43.9)
Female		72 (52.2)		23 (56.1)
Age	55.1 (10.1)		10.2 (3.5)	
BMI/BMIz ^1^	36.0 (5.8)		2.4 (0.4)	
Missing		5		
Weight status				
Overweight		18 (13.5)		3 (7.3)
Obesity		44 (33.1)		23 (56.1)
Severe obesity		45 (33.8)		11 (26.8)
Morbid obesity		26 (19.5)		4 (9.8)
Missing		5		
BMI of mother ^1^			30.4 (6.5)	
Missing				7
BMI of father ^1^			27.3 (4.6)	
Missing				14
Country of birth				
The Netherlands		125 (95.4)		35 (97.2)
Other		6 (4.6)		1 (2.8)
Missing		7		5
Ethnicity ^2^				
Dutch				29 (74.4)
Turkish				2 (5.1)
Moroccan				8 (20.5)
Missing				2
Educational level of adults or of mother				
Low		50 (38.2)		11 (29.7)
Intermediate		53 (40.5)		16 (43.2)
High		28 (21.4)		10 (27.0)
Missing		7		4
Educational level of father				
Low				21 (56.8)
Intermediate				9 (24.3)
High				7 (18.9)
Missing				4
Working situation (of parents)				
Not in work		67 (51.1)		6 (15.4)
One parent in work				13 (33.3)
In work		64 (48.9)		20 (51.3)
Missing		7		2
Living situation (of parents)				
Living alone		38 (28.8)		7 (17.9)
Living together		94 (71.2)		32 (82.1)
Missing		6		2
Family size			4.2 (1.1)	
Missing				2

Notes: SD = standard deviation, *N* = number of participants; ^1^ in kg/m^2^ or standardized BMI, ^2^ based on the children’s and parents’ country of birth.

**Table 2:** Average changes among adults regarding psychological needs, motivation for physical activity and healthy diet, barriers, physical activity, dietary behaviour, quality of life and body mass index (BMI), at T1 and T2 compared to T0, analysed using linear mixed models.

Outcome Variable	Pre-Test (T0)	Change T1–T0		Change T2–T0	
	Mean (SD)	B (CI)	Effect Size	B (CI)	Effect Size
Psychological needs ^1^					
Autonomy	4.3 (0.7)	0.2 (0.0, 0.3) **	0.31	0.2 (0.1, 0.3) **	0.40
Competence	3.3 (0.9)	0.2 (0.0, 0.4)	0.27	0.2 (−0.1, 0.4)	0.29
Relatedness	3.4 (1.0)	−0.1 (−0.3, 0.1)	0.04	−0.1 (−0.4, 0.1)	−0.04
Motivation for physical activity ^2^					
Amotivation	0.6 (0.7)	0.0 (−0.1, 0.2)	−0.01	0.0 (−0.2, 0.2)	−0.01
External regulation	0.9 (1.1)	−0.3 (−0.4, −0.1) **	−0.24	−0.3 (−0.5, −0.2) ***	−0.28
Introjected regulation	1.4 (1.0)	0.0 (−0.2, 0.2)	0.05	−0.2 (−0.4, 0.1)	−0.14
*Controlled motivation* ^3^	5.0 (3.9)	−0.4 (−1.2, 0.3)	−0.14	−0.8 (−1.6, 0.0) *	−0.21
Identified regulation	2.8 (0.9)	0.1 (−0.1, 0.3)	0.16	0.0 (−0.2, 0.1)	0.00
Integrated regulation	2.2 (1.1)	0.3 (0.1, 0.5) ***	0.30	0.2 (0.0, 0.4)	0.14
Intrinsic motivation	2.5 (1.2)	0.3 (0.1, 0.5) ***	0.36	0.2 (0.0, 0.4) *	0.16
*Autonomous motivation* ^4^	14.7 (6.3)	1.9 (0.9, 2.9) ***	0.33	1.0 (0.0, 1.9)	0.14
Motivation for healthy diet ^2^					
Amotivation	0.6 (0.8)	0.0 (−0.2, 0.1)	−0.06	0.0 (−0.1, 0.2)	0.04
External regulation	2.0 (1.0)	−0.2 (−0.4, −0.1) *	−0.16	−0.2 (−0.4, 0.0)	−0.15
Introjected regulation	2.1 (1.0)	−0.2 (−0.4, 0.0) *	−0.14	−0.2 (−0.4, 0.0) *	−0.19
*Controlled motivation* ^3^	8.0 (3.9)	−0.9 (−1.5, −0.2) *	−0.21	−0.5 (−1.3, 0.3)	−0.10
Identified regulation	3.6 (0.6)	0.0 (−0.2, 0.1)	−0.01	−0.1 (−0.2, 0.1)	−0.09
Integrated regulation	2.6 (1.0)	0.4 (0.2, 0.6) ***	0.52	0.3 (0.1, 0.5) **	0.34
Intrinsic motivation	2.9 (0.9)	0.2 (0.1, 0.4) ***	0.38	0.0 (−0.1, 0.2)	0.10
*Autonomous motivation* ^4^	17.5 (4.6)	1.6 (0.9, 2.2) ***	0.46	0.7 (−0.2, 1.5)	0.19
Barriers ^1^					
Physical activity	2.5 (0.8)	−0.3 (−0.5, −0.2) ***	−0.52	−0.3 (−0.4, −0.2) ***	−0.44
Eating a healthy diet	2.1 (0.8)	−0.3 (−0.5, −0.2) ***	−0.51	−0.3 (−0.5, −0.1) ***	−0.44
Physical activity					
Sedentary behaviour ^5^	437.1 (198.8)	−123.2 (−162.2, −84.2) ***	−0.69	−102.9 (−151.6, −54.2) ***	−0.52
Walking ^6^	284.3 (327.9)	−35.3 (−101.4, 30.7)	−0.15	−12.1 (−83.2, 59.0)	−0.06
Moderate-intensity activities ^6^	229.6 (326.5)	72.2 (−23.3, 167.7)	0.25	106.8 (16.5, 197.0) *	0.32
Vigorous-intensity activities ^6^	96.2 (167.7)	78.2 (26.3, 130.0) **	0.41	87.5 (25.4, 149.5) **	0.50
*Total*	593.8 (662.6)	131.6 (−22.6, 285.8)	0.18	202.0 (37.9, 366.1) *	0.28
Dietary behaviour					
Breakfast ^7^	6.3 (1.8)	0.5 (0.2, 0.8) **	0.24	0.2 (−0.1, 0.5)	0.11
Fruits ^8^	9.4 (7.6)	1.8 (0.3, 3.2) *	0.26	0.8 (−0.6, 2.1)	0.21
Vegetables ^9^	26.8 (12.4)	2.4 (0.0, 4.7)	0.21	3.0 (−0.9, 6.9)	0.18
Fruit juices ^7^	2.1 (2.3)	−0.7 (−1.1, −0.3) ***	−0.33	−0.5 (−1.0, 0.0)	−0.28
Sugar-sweetened beverages ^7^	2.7 (2.9)	−1.0 (−1.5, −0.6) ***	−0.44	−1.0 (−1.5, −0.5) ***	−0.40
Unhealthy snacks ^7^	8.7 (6.3)	−2.5 (−3.6, −1.5) ***	−0.33	−1.5 (−2.9, −0.1) *	−0.17
Quality of life					
Mobility ^10^	1.5 (0.5)	−0.1 (−0.2, 0.0)	−0.16	0.0 (−0.1, 0.0)	−0.24
Self-care ^10^	1.1 (0.3)	−0.1 (−0.1, 0.0) *	−0.20	0.0 (−0.1, 0.1)	−0.07
Usual activities ^10^	1.4 (0.5)	0.0 (−0.1, 0.0)	−0.11	−0.1 (−0.2, 0.1)	−0.19
Pain/discomfort ^10^	1.8 (0.6)	−0.1 (−0.2, 0.0) *	−0.19	−0.1 (−0.2, 0.0) *	−0.29
Anxiety/depression ^10^	1.5 (0.6)	−0.2 (−0.3, −0.1) ***	−0.37	−0.2 (−0.3, −0.1) **	−0.35
*Total* ^11^	0.7 (0.3)	0.1 (0.0, 0.1) **	0.24	0.1 (0.0, 0.1) *	0.32
In general ^12^	6.3 (1.4)	0.5 (0.2, 0.7) ***	0.42	0.7 (0.4, 0.9) ***	0.57
At that moment ^12^	6.5 (1.3)	0.3 (0.0, 0.5)	0.22	0.5 (0.2, 0.8) **	0.42
Weight and BMI					
Weight ^13^	105.4 (19.7)	−2.3 (−3.3, −1.3) ***	−0.13	−1.8 (−2.8, −0.8) ***	−0.11
BMI ^14^	36.2 (5.9)	−0.8 (−1.1, −0.4) ***	−0.13	−0.6 (−0.9, −0.3) ***	−0.14

Notes: Results of linear mixed model analyses (unstructured covariance type); analyses were controlled for background characteristics if they were significantly related to missingness: age, gender, living situation, occupational status, highest completed education and BMI; SD = standard deviation, B is the corrected estimated mean difference in change scores from baseline, CI = confidence interval, significantly different (* *p* < 0.050; ** *p* < 0.010; *** *p* < 0.001); ^1^ five-point scale (1 = totally disagree; 5 = totally agree), ^2^ five-point scale (0 = totally disagree; 4 = totally agree), ^3^ sum score (0 = uncontrolled motivation; 24 = controlled motivation), ^4^ sum score (0 = no autonomous motivation; 24 = autonomous motivation), ^5^ outcome measured in minutes per day, ^6^ minutes per week, ^7^ frequency per week, ^8^ pieces per week, ^9^ serving spoons per week, ^10^ three-point scale (1 = no disability; 3 = greatly disabled), ^11^ correction factor (−0.33 = completely unhealthy; 1 = completely healthy), ^12^ grade (1–10); ^13^ in kg; ^14^ in kg/m^2^.

**Table 3:** Average change reported by parents and children regarding parenting, motivation for physical activity and healthy diet, behaviour-specific cognitions, physical activity, dietary behaviour, sleeping behaviour, quality of life and BMIz, at T1 and T2, analysed with linear mixed models.

Outcome Variable	Pre-Test (T0)	Change T1–T0		Change T2–T0	
	Mean (SD)	B (CI)	Effect Size	B (CI)	Effect Size
General parenting					
Monitoring ^1,a^	3.4 (1.0)	0.0 (−0.6, 0.7)	0.12	−0.1 (−0.7, 0.5)	−0.01
Consistent discipline ^1,a^	2.8 (1.3)	0.2 (−0.3, 0.8)	0.19	0.3 (−0.4, 1.1)	0.28
Autonomy support ^1,a^	4.1 (1.0)	−0.1 (−0.6, 0.3)	0.01	0.2 (−0.3, 0.7)	0.18
Problem scale ^2^	2.7 (1.1)	−0.5 (−0.8, −0.1) *	−0.24	−0.3 (−0.8, 0.2)	−0.23
Confidence scale ^3,b^	6.6 (2.3)	1.3 (0.3, 2.4) **	0.74	0.4 (−0.9, 1.8)	0.22
Parenting practices					
Stimulation to be active and to eat a healthy diet ^1^	4.4 (0.5)	0.1 (−0.1, 0.3)	0.15	0.0 (−0.2, 0.3)	0.13
Parental role modelling—healthy eating ^1^	4.0 (0.6)	0.3 (0.1, 0.6) **	0.51	0.3 (0.0, 0.5) *	0.59
Parental role modelling—physical activity ^4,a^	3.1 (0.6)	0.4 (0.2, 0.6) ***	0.66	0.1 (−0.1, 0.3)	0.19
Food environment ^1,a^	3.5 (1.0)	0.4 (0.0, 0.8) *	0.35	0.5 (0.1, 1.0) *	0.65
Parental policies ^4,a^	3.9 (0.8)	0.1 (−0.1, 0.4)	0.35	−0.1 (−0.4, 0.2)	−0.06
Emotional feeding ^4^	1.3 (0.5)	0.0 (−0.1, 0.2)	−0.03	0.2 (−0.1, 0.5)	0.32
Instrumental feeding ^4^	1.5 (0.6)	0.0 (−0.1, 0.2)	−0.22	0.0 (−0.2, 0.3)	0.15
Monitoring physical activity ^4^	3.7 (0.7)	0.2 (0.0, 0.3)	0.30	−0.2 (−0.5, 0.2)	−0.25
Covert control ^4^	2.9 (0.9)	0.3 (0.0, 0.6) *	0.35	0.3 (0.0, 0.7) *	0.48
Child control ^4^	2.4 (0.7)	0.2 (−0.1, 0.5)	0.22	0.1 (−0.2, 0.3)	0.07
Motivation for physical activity ^1^					
Amotivation ^a^	0.8 (1.2)	−0.1 (−0.5, 0.3)	0.00	−0.3 (−0.8, 0.2)	−0.12
External regulation ^b^	0.8 (0.7)	−0.1 (−0.8, 0.6)	−0.21	0.0 (−0.5, 0.4)	−0.09
Introjected regulation	1.0 (0.6)	0.2 (−0.1, 0.4)	0.13	−0.2 (−0.6, 0.1)	−0.38
*Controlled motivation* ^5^	5.2 (5.0)	0.5 (−1.4, 2.5)	−0.11	−0.4 (−3.4, 2.7)	−0.19
Identified regulation	2.3 (1.0)	−0.2 (−0.9, 0.4)	−0.03	0.1 (−0.4, 0.5)	0.27
Intrinsic motivation	2.7 (1.1)	0.1 (−0.3, 0.6)	0.48	0.0 (−0.6, 0.6)	0.21
*Autonomous motivation* ^6^	12.7 (5.2)	−1.1 (−2.7, 0.4)	0.30	−0.4 (−3.2, 2.4)	0.24
Behaviour-specific cognitions					
Physical activity enjoyment ^1^	4.0 (0.8)	0.0 (−0.3, 0.3)	−0.18	−0.1 (−0.3, 0.1)	−0.14
Habit strength—sports ^1^	3.5 (1.0)	−0.1 (−0.5, 0.4)	−0.29	−0.1 (−0.4, 0.3)	−0.18
Habit strength—playing outside ^1^	3.3 (1.1)	0.2 (−0.3, 0.7)	0.38	−0.1 (−0.7, 0.5)	0.23
Habit strength—eating fruits ^1^	3.2 (1.1)	0.1 (−0.2, 0.4)	0.42	0.4 (−0.2, 1.0)	0.40
Self-efficacy—playing outside ^7^	2.9 (1.0)	−0.1 (−0.6, 0.3)	−0.23	0.1 (−0.5, 0.6)	0.41
Self-efficacy—eating fruits ^7^	3.2 (0.6)	−0.1 (−0.4, 0.2)	−0.26	0.0 (−0.4, 0.3)	0.09
Attitude—eating fruits ^1^	4.1 (0.6)	−0.3 (−0.5, 0.0) *	−0.42	−0.2 (−0.4, 0.1)	−0.20
Physical activity ^8^					
Watching television	9.8 (5.9)	−2.1 (−4.0, −0.2) *	−0.23	−4.5 (−6.6, −2.4) ***	−0.91
Using a computer	9.2 (7.2)	0.5 (−2.0, 3.0)	0.10	1.7 (−0.9, 4.3)	0.28
Playing outside	7.4 (6.7)	−1.1 (−3.4, 1.2)	−0.10	−1.8 (−4.8, 1.1)	−0.29
Engaging in sports	3.1 (3.6)	0.4 (−0.8, 1.6)	0.00	−0.4 (−1.4, 0.7)	−0.20
Walking or biking to school	3.0 (3.9)	0.6 (−0.3, 1.5)	−0.08	0.2 (−1.4, 1.9)	0.09
Walking or biking during leisure time	4.6 (5.7)	−0.2 (−2.2, 1.9)	−0.10	−1.4 (−3.4, 0.5)	−0.27
Sports behaviour ^9,b^	3.0 (1.5)	−0.9 (−2.2, 0.5)	−0.22	1.5 (−0.3, 3.2)	0.66
Dietary behaviour					
Breakfast ^9^	6.0 (1.8)	0.5 (−0.1, 1.1)	0.24	0.0 (−0.4, 0.4)	0.08
Fruits ^10^	9.3 (6.3)	2.8 (−2.3, 8.0)	0.58	0.6 (−1.5, 2.7)	0.03
Vegetables ^11,b^	14.5 (9.9)	6.6 (0.8, 12.3) *	0.68	5.9 (−1.1, 13.0)	0.92
Water ^12^	16.2 (16.8)	12.7 (3.2, 22.1) *	0.61	8.3 (1.4, 15.3) *	0.27
Sugar-sweetened beverages ^12^	8.3 (11.2)	−3.9 (−11.0, 3.3)	−0.23	−5.6 (−10.5, −0.8) *	−0.55
Diet beverages ^12^	2.7 (5.3)	2.7 (2.7, 8.0)	0.61	−0.5 (−2.6, 1.5)	−0.09
Fruit beverages ^12^	1.9 (2.8)	−0.4 (−3.0, 2.3)	−0.10	−1.2 (−2.4, 0.0) *	−0.47
Fruit juices ^12^	1.6 (2.4)	−1.1 (−2.0, −0.2) *	−0.40	−1.0 (−2.0, 0.0)	−0.41
Energy drinks ^12,b^	0.1 (0.5)	0.1 (−0.2, 0.4)	0.19	0.0 (−0.3, 0.3)	−0.05
Unhealthy snacks ^9^	8.3 (4.2)	−0.9 (−2.6, 0.7)	−0.33	−0.8 (−2.1, 0.5)	−0.17
Sleeping behaviour					
Sleeping hours ^8^	48.5 (7.8)	1.2 (−2.4, 4.8)	0.16	0.5 (−3.3, 4.3)	0.12
Sleeping problems ^13^	0.3 (0.7)	0.0 (−0.3, 0.4)	0.22	−0.1 (−0.4, 0.2)	−0.13
Quality of life					
Physical comfort ^14^	25.2 (5.3)	1.9 (0.2, 3.5) *	0.25	1.4 (−0.6, 3.4)	0.35
Body esteem ^14^	35.4 (8.2)	1.8 (−0.7, 4.2)	0.06	0.0 (−3.0, 3.1)	0.01
Social life ^14^	26.0 (4.4)	0.4 (−0.8, 1.6)	0.07	0.8 (−0.3, 1.8)	0.14
Family relations ^14^	28.3 (2.4)	0.8 (−0.3, 1.9)	0.35	0.7 (−0.5, 1.8)	0.18
*Total* ^15^	115.2 (16.5)	4.4 (0.8, 8.1) *	0.16	2.2 (−3.4, 7.7)	0.15
Health of parent in general ^3^	7.6 (1.0)	−0.1 (−0.5, 0.3)	−0.13	−0.2 (−0.6, 0.3)	−0.05
Health of parent at that moment ^3^	7.5 (1.1)	0.0 (−0.6, 0.7)	−0.07	−0.2 (−0.7, 0.4)	−0.10
Health of child in general ^3^	7.4 (1.5)	0.3 (−0.1, 0.8)	0.22	−0.1 (−0.6, 0.4)	0.08
Health of child at that moment ^3^	7.4 (1.6)	0.5 (0.0, 1.1)	0.31	−0.1 (−0.6, 0.4)	0.13
BMIz					
BMIz ^16^	2.4 (0.4)	−0.1 (−0.2, 0.0)	−0.24	0.0 (−0.1, 0.1)	−0.06

Notes: Results of linear mixed model analyses (unstructured covariance type); analyses were controlled for background characteristics if they were significantly related to missingness: age, gender, living situation, occupational status, highest completed education and BMIz; SD = standard deviation, B is the corrected estimated mean difference in change scores from baseline, CI = confidence interval, significantly different (* *p* < 0.050; ** *p* < 0.010; *** *p* < 0.001), ^a^ 1-item analysis, ^b^ compound symmetry analysis; ^1^ five-point scale (1 = totally disagree; 5 = totally agree), ^2^ seven-point scale (1 = no problem at all; 7 = very much a problem), ^3^ grade (1–10), ^4^ five-point scale (1 = never; 5 = always), ^5^ sum score (0 = uncontrolled motivation; 24 = controlled motivation), ^6^ sum score (0 = no autonomous motivation; 20 = autonomous motivation), ^7^ five-point scale (1 = very hard; 5 = not hard at all), ^8^ hours per week, ^9^ frequency per week, ^10^ pieces per week, ^11^ serving spoons per week, ^12^ cups per week, ^13^ five-point scale (0 = no problems; 1 = 1 problem; 4 = 4 problems), ^14^ five-point scale (1 = always true; 5 = never true), ^15^ scale (1 = worse; 135 = best), ^16^ standardized BMI.

## Appendix A. Additional Tables

**Table A1:** Techniques and approaches used in the Coaching on Lifestyle (CooL) intervention.

Techniques, Key Terms and Approaches Used in the Adult Programme	Techniques and Approaches Used in the Children and Adolescent Programme *All techniques and approaches used for the adult programme are combined with specific interventions, because of the specific nature of the educational relationship between parents and their children*
Autonomy-supportive coaching	General parenting constructs (control, responsiveness and structure)
Celebrating (small) successes	
Coaches providing feedback and compliments	Parenting practices (e.g., modelling, rule setting and rewarding) to stimulate desired behaviour and to restrict undesired behaviour
Communication skills of the client	
Dealing with lapses/relapses and temptations	
Estimating the value of (different sources of) information	Negotiation skills
Goal setting	
Implementation intentions	
Increasing self-efficacy	
Knowledge about healthy lifestyle and behavioural change	
Mobilizing social support	
Motivational interviewing	
Nudging	
Ownership	
Peer modelling, peer support and peer learning	
Positive health and positive psychology	
Rewarding	
Self-management, self-monitoring and self-evaluation	
Stepping out of one’s comfort zone	

**Table A2:** Constructs used in the questionnaires for the adult participants, with their corresponding scales.

Construct	Scale	Items	Answering Options	Cronbach’s Alpha
**Personal factors**				
*Demographics*	-	9	Divers	-
*Psychological needs*	Psychological Need Satisfaction in Exercise scale (PNSE) [54]	18	Totally disagree/totally agree ^1^	
Autonomy		6		0.9
Competence		6		0.9
Relatedness		6		0.9
**Motivation**				
*Quality of motivation for physical activity*	Behavioural Regulation in Exercise Questionnaire (BREQ-3) [55,56]	23	Totally disagree/totally agree ^1^	
Amotivation (AM)		4		0.7
External regulation (EX)		4		0.8
Introjected regulation (IJ)		3		0.7
Controlled motivation			Sum score = (AM*3) + (EX*2) + (IJ*1)	
Identified regulation (ID)		4		0.7
Integrated regulation (IG)		4		0.9
Intrinsic motivation (IM)		4		0.9
Autonomous motivation			Sum score = (ID*1) + (IG*2) + (IM*3)	
*Quality of motivation for healthy diet*	Regulation of Eating Behaviour Scale (REBS) [57]	24	Totally disagree/totally agree ^1^	
Amotivation		4		0.7
External regulation		4		0.8
Introjected regulation		4		0.7
Controlled motivation			Sum score = (AM*3) + (EX*2) + (IJ*1)	
Identified regulation		4		0.7
Integrated regulation		4		0.8
Intrinsic motivation		4		0.8
Autonomous motivation			Sum score = (ID*1) + (IG*2) + (IM*3)	
**Behaviour-specific cognitions**				
*Barriers*				
For physical activity		11	Totally disagree/totally agree ^1^	0.8
For eating a healthy diet		10	Totally disagree/totally agree ^1^	0.8
**Energy balance-related behaviours**				
*Physical activity*	International Physical Activity Questionnaire (IPAQ)	7	Days per week, minutes per day	
Sedentary behaviour		1		
Walking		2		
Moderate-intensity activities		2		
Vigorous-intensity activities		2		
*Dietary behaviours*	Shortened Fat List [58]	20	Days per week, frequency per day	
Breakfast		1		
Fruits		2		
Vegetables		4		
Fruit juices		1		
Sugar-sweetened beverages		1		
Unhealthy snacks		7		
**Quality of life and BMI**	-			
*Quality of life*	EQ-5D-3L [59]	7	No disability/strongly disabled ^2^	
Mobility		1		
Self-care		1		
Usual activities		1		
Pain/discomfort		1		
Anxiety/depression		1		
Total quality of life	[60]		Index = 1 (complete health—0.071 (if there is a problem on a dimension)—deduction per problem on a dimension	
In general		1		
At that moment		1		
*BMI*	[32]	2	Centimetres, kilograms	

Notes: ^1^ Five-point scale, ^2^ three-point scale.

**Table A3:** Constructs used in the questionnaires for the children and their parents, with their corresponding scales.

Construct	Scale	Items	Answering Options	Cronbach’s Alpha	Group
**Personal factors**					
*Demographics*	-	16	Divers	-	Parent
**General parenting**					
*General Parenting*	Shortened version of the Comprehensive General Parenting Questionnaire (CGPO) [61]	3	Totally disagree/totally agree ^1^		Parent
Monitoring		1			
Consistent discipline		1			
Autonomy support		1			
*Children’s behavioural problems and parents’ self-efficacy*	Lifestyle Behaviour Checklist (LBC) [62,63]	50			Parent
Problem scale		25	Not at all/very much ^2^	0.9	
Confidence scale		25	Grade ^3^	1.0	
**Parenting practices**					
*Stimulation to be active and to eat a healthy diet*	[64]	5	Totally disagree/totally agree ^1^	0.6	Parent
*Parental role modelling—healthy eating*	Comprehensive Feeding Practices Questionnaire (CFPQ) [65]	4	Totally disagree/totally agree ^1^	0.6	Parent
*Parental role modelling—physical activity*	Home Environment Survey (HES) [66]	7	Totally disagree/totally agree ^1^	0.6	Parent
*Food environment*	CFPQ [65]	2	Never/always ^1^	0.8	Parent
*Parental policies*	HES [66]	2	Never/always ^1^	0.6	Parent
*Emotional feeding*	Parental Feeding Style Questionnaire (PFSQ) [67]	5	Never/always ^1^	0.8	Parent
*Instrumental feeding*	PFSQ [67]	4	Never/always ^1^	0.7	Parent
*Monitoring physical activity*	Physical activity [64] and dietary intake with Child Feeding Questionnaire (CFQ) [68]	6	Never/always ^1^	0.8	Parent
*Covert control*	Covert Control Scale [69]	3	Never/always ^1^	0.7	Parent
*Child control*	CFPQ [65]	5	Never/always ^1^	0.7	Parent
**Motivation**					
*Motivation for physical activity*	Behavioural Regulation of Physical Activity in Children (BRePAC) [70]	18	Totally disagree/totally agree ^1^		Child
Amotivation		1			
External regulation		4		0.6	
Introjected regulation		5		0.5	
Controlled motivation			Sum score = (AM*3) + (EX*2) + (IJ*1)		
Identified regulation		5		0.8	
Intrinsic motivation		2		0.7	
Autonomous motivation			Sum score = (ID*2) + (IM*3)		
**Behaviour-specific cognitions**					
*Physical activity enjoyment*	Physical Activity Enjoyment Scale (PACES) [71]	16	Totally disagree/totally agree ^1^	0.9	Child
*Habit strength*	Shortened version of the Self-Report Habit Index (SRHI) [72]	9	Totally disagree/totally agree ^1^		Child
Sporting		2		0.6	
Playing outside		2		0.9	
Eating fruits		2		0.7	
*Self-efficacy*	[73]	21	Very difficult/totally not difficult ^1^		Child
Playing outside		13		0.9	
Eating fruits		8		0.6	
*Attitude about eating fruits*		7	Totally disagree/totally agree ^1^	0.6	Child
**Energy balance-related behaviours**					
*Physical activity*	[74]	14	Not at all/3 h or longer ^4^, I don’t know		Parent
Watching television		2			
Using a computer		2			
Playing outside		2			
Engaging in sports		2			
Walking or biking to school		2			
Walking or biking during leisure time		2			
Sports behaviour		1	Days per week		Child
*Dietary behaviours*	[75]	26	Days per week, frequency per day		Parent
Breakfast		1			
Fruits		2			
Vegetables		4			
Water		2			
Sugar-sweetened beverages		2			
Diet beverages		2			
Fruit beverages		2			
Fruit juices		2			
Energy drinks		2			
Unhealthy snacks		7		0.7	
*Sleeping behaviour*	[76]	6			Parent
Sleeping hours		2	Hours per day		
Sleeping problems		4	Never/always ^1^		
**Quality of life and BMIz**					
*Quality of life*	Impact of Weight on Quality of Life (IWQOL)-Kids [77,78]	31	Never/always ^1^		Parent
Physical comfort		6		0.9	
Body esteem		9		0.9	
Social life		6		0.9	
Family relations		6		0.7	
Total quality of life			Sum score		
Health of parent in general		1			
Health of parent at that moment		1			
Health of child in general		1			
Health of child at that moment		1			
*BMIz*	[28,33,34]	4	Centimetres, kilo-grams, age, ethnicity		Parent

Notes: ^1^ Five-point scale, ^2^ seven-point scale, ^3^ ten-point scale, ^4^ six-point scale.
